# Distributed Response to Distributed Intervening: Making Sense of Public Digitalization Through Digital Support

**DOI:** 10.1007/s10912-025-09982-1

**Published:** 2025-09-06

**Authors:** Stig Bo Andersen, Sofie Skovbæk, Aske Juul Lassen, Astrid Pernille Jespersen

**Affiliations:** Faculty of Humanities, Saxo Institute, Copenhagen Centre for Health Research in the Humanities, University of Copenhagen, Copenhagen, Denmark

**Keywords:** Digitalization, Digital citizenship, Denmark, Distributed agency, Response-ability, Medical humanities

## Abstract

Communication and interaction with public authorities and healthcare professionals in Denmark primarily go through digital self-service platforms, requiring diverse skills and device access. In this article, we describe how senior citizens in Denmark handle and make sense of public digitalization through different forms of digital support. Through an ethnographic study of community-led initiatives of digital support, we highlight how senior citizens find socio-technical ways of managing digital obligations and argue that citizens’ digital agency in day-to-day interactions with public digitalization relies heavily on distributed socio-material relations. We suggest that the ways of engaging with healthcare through digital means should be of increasing concern to medical humanities scholars, as digital literacies and technologies have become gatekeepers to welfare and healthcare. Drawing on Donna Haraway’s reconceptualization of responsibility as response-ability, and Jane Bennett’s notion of distributed agency, we argue that the ability of digital citizens to respond is a result of a distributed and combined responsiveness of human, technological, and digital actants. We point to two opposite but interrelated assemblages: *public digital as distributed intervening* mediated through computers, smartphones, tablets, public digital mail platforms, et cetera, and *digital support as distributed response*, which serves to mitigate and translate demands and obligations of the digitalized welfare state. Consequently, as digital developments tend to generate an increasingly individualizing gaze, medical humanities must be critically concerned with the manifold, subtle actants that co-constitute accessibility and responsiveness of patients and citizens.

## Introduction

In mid-2023, the Danish government announced that the next chapter of Danish digital development is to become world-leading in digital inclusion. To quote a governmental vision statement: “Being a welfare state built on values of inclusion, the welfare state should also embrace those with limited digital capabilities and provide the necessary support, tools, and alternatives” (authors translation, Ministry of Digital Government and Gender Equality 2023, 6). The Danish digitalization of the public sector has developed into a massive field of state and private stakeholders that provide citizens with digitalized access to all corners of the public sector services, such as healthcare, social benefits, tax, et cetera, through a wide range of digital self-service platforms, in-person citizen service centers, a personal two-factor log-in authenticator, and public digital mail providers. However, the political acknowledgment of the abundance of new digital and social practices and the constraints of the citizens’ digital everyday doings when communicating with the digitalized public sector remains scarce.

In this article, we argue for the need to decenter the individual responsibility of the citizen, politically framed as a self-dependent actor in discourses of public digitalization and digital interactions with the Danish welfare state (Hjelholt and Schou 2017; Pors and Schou 2021). Our argument is based on an ethnographic study of digital support opportunities for older adults in three municipalities of Denmark, which we conducted in the fall of 2022 and spring of 2023. We aim to show how being able to respond to—and with—public digitalization in day-to-day interactions relies on the distributed agency of citizens, hardware, software, peers, and civil servants. In this regard, we highlight how there is an important divergence between the digital practices of the older adults and the laws and regulations directing such practices.

The digitalization of public welfare is of increasing relevance to medical humanities, as access to healthcare, medical information, and medical professionals’ practices in a Nordic context has become entangled with various digital platforms for patients to access their medical information and communicate with professionals and authorities within welfare. In Denmark, citizens can access their medical journals and test results online, make appointments with their general practitioner (although this can still be done via telephone as well), and find information about hospital appointments in their digital mailbox provided by the state. These services can only be accessed via a two-factor login. As medical humanities has sought to widen our understanding of health and medicine for decades, the field has seen a continuous discussion and expansion of areas of interest (ex. Atkinson et al. 2015; Brody 2011; Charise 2017; Whitehead and Woods 2016; Viney et al. 2015). Recently, Pietrzak-Franger (2023) has called for furthering the understanding of *postdigital health practices* as new directions for medical humanities, diagnosing a decreasing infatuation with digital development. Pietrzak-Franger argues that digital technologies have become mundane and ubiquitous in our everyday lives and, thus, have become co-constituents of our lives. This emphasis may be most prominent in the related field of Socio-Gerontechnology, which takes the co-constitution of aging and technology as its point of departure (Peine et al. 2015; 2021; Peine and Neven 2019; Wanka and Gallistl 2018). As nearly all encounters with the Danish public welfare system have become digitized with access to welfare services being largely facilitated through smartphones and computers, we argue that digitalization, practices of digital support, and access to healthcare via digital platforms are increasingly of relevance to the field of medical humanities.

### Danish developments of public digitalization

Building on neoliberal ideals of individual freedom, Danish digital development has sought to liberate the citizens from in-person bureaucratic queues while simultaneously reducing the costs of public administration (Schou and Hjelholt 2018). Consequently, citizens are expected to be self-dependent and autonomous in their daily digital doings within a wide range of self-service solutions, public digital mail platforms, healthcare platforms, and two-factor authentication login. As Denmark, according to the UN, has the most digitalized public sector in the world (United Nations 2022), it is already a frontier of both digital e-governance and of said developments’ successes and shortcomings.

A long list of skills and literacies is needed to be digitally self-sufficient, with digital and administrative literacies as major factors (Agency for Digital Government 2023; Döring 2021); 17.2% of the Danish population have insufficient digital and/or administrative competencies, with 62% of these so-called “digitally challenged” being above 65 years old (Agency for Digital Government 2023). One out of four above 75 years experiences that digital development impedes their independence (Epinion 2023). Several organizations representing marginalized groups, including the nationwide organization of senior citizens, Dane Age, have criticized the Danish digital development, urging the government to recognize and include the needs of citizens with insufficient digital skills and literacies (DaneAge 2022).

Citizens’ ability to respond to public digital interactions within the digitalized Nordic welfare states has consequences regarding citizens’ access to health. While some of the examples we use in the article do not directly relate to health, the interlocutors do not distinguish between digital struggles related to health and digital struggles related to other spheres of the welfare state. As digital literacies gradually become a gatekeeper to welfare and healthcare, digital inclusion increasingly becomes a “super social determinant of health” as it addresses all other social determinants of health (Sieck et al. 2021). Citizens with insufficient digital skills are arguably to be understood as a certain kind of *neoliberal misfits* as they experience the debilitating flipside of the forms of subjectivity celebrated by neoliberal discourse (Skiveren 2022, 603). Medical humanities, we argue, should address these issues of inequality of digital access to healthcare.

Few Danish and international studies have discussed how citizens seek to mitigate growing expectations within changing digital platforms, with *formal* digital support given by authorities and public institutions on one side (in a Danish context: Carreras and Finken 2022; Pors and Schou 2021; Schou and Hjelholt 2018) and *informal* support within families and between close relations on the other (Agency for Digital Government 2023). Studies within the field of socio-gerontechnology have especially underlined the effects of informal types of help to both educate and keep older adults up to date with new technologies (Tsai et al. 2017; Rosales and Blanche-T 2022; Hunsaker et al. 2019; Kuoppamäki et al. 2022). In our study, we have identified a third, *nonformal* category of digital support, referring to situations of support that are continuous efforts of digital support within community settings with varying degrees of organization (Andersen et al. 2024). With the prefix “non” in nonformal, we wish to highlight how this category of digital support is neither strictly formal and regulated nor is it strictly informal and unregulated. Rather, the employees (e.g., healthcare professionals and social workers) conducting this kind of support are not employed in order to provide digital support, and often, they are not formally allowed to engage in this kind of support. Also, the volunteers engaged in this kind of support work according to overall guidelines but often need to bypass such guidelines in order to provide adequate support. In this article, we focus on nonformal support practices.

## Material and methods

This study is part of a collaborative outreach project on the diverse transition processes from work life to senior life. The initial outreach project was partly carried out during the COVID-19 pandemic, with many project activities becoming online activities. Simultaneously, the new personal public digital login authenticator, MitID, was rolled out in Denmark, which came with a plethora of challenges, especially for citizens with limited digital skills or without updated technology. These two experiences made us realize the importance of digital development in senior life, and they highlighted the heterogeneous ways in which the project participants related to digitalized communication negatively and positively. We therefore decided to design a subproject on the everyday digital interactions of older adults and how, when, and where they sought support when confronted with a digital challenge.

We carried out an ethnographic study from the fall of 2022 to the spring of 2023 in one rural and two major cities in Denmark focusing on practices and needs of digital support of citizens aged 55 and older. In total, we conducted 37 semi-structured interviews categorized as either digital supporters (*N* = 30) or supported persons (*N* = 25). The digital supporters consisted of a broad spectrum of people that were regularly in situations of giving digital support. They ranged from publicly employed authorities at citizen service centers, librarians, social workers, and health professionals to privately employed IT-educators and volunteer digital supporters. The digital supporters varied in age, but volunteer digital supporters were predominantly in the same age group as the digitally supported persons in this study. The supported persons were citizens aged 55 and above, who were initially interviewed regarding their need for support when using public digital services.

A multitude of methods were used. One-on-one interviews and group interviews were used to explore the practices of digital support, with one interview conducted online, while participatory observations and informal conversations (*N* = 61) were carried out in multiple public and community-led sites of digital support targeting older adults to study the situated practices of digital support. At these sites, we were provided with the opportunity to observe how the supporting and supported persons used computers, smartphones, and printers, etc., and thus how the agency was distributed between citizens, materialities, and the welfare state. In total, this study relies on the statements of 116 interlocutors and their devices.

In recruiting interlocutors, we took an open-minded approach to explore the width of sites of digital support, reaching out to activities we found online and taking advantage of a “snowball effect” of interlocutors pointing us to sites of digital support. Simultaneously, we interviewed older adults on their use of digital support, which we recruited primarily through our municipality partners of the aforementioned initial outreach project or through the activities mentioned above. In our study, sites of digital support came to be places where citizens met peers, authorities, healthcare professionals, or social workers and where exchanges of knowledge or guidance on public digital platforms were common (and sometimes subtle). The examples used in this article are selected to highlight a width of situated social dynamics of people and devices when engaging in situations of digital support, and how supporters, the supported, and their devices work in tandem.

While keeping interviews open-ended and exploratory, we constructed two interview guides: one for the older adults focusing on everyday digital interactions with public digital platforms and where they sought support, and the other for digital supporters focusing on their situated practices of doing digital support. As it played out, the fixed definitions of supported and supporter were difficult to maintain, as most of our interlocutors had experiences of giving and receiving digital support and guidance.

Interviews were recorded and observations were written down as field notes immediately after leaving the observations. Interviews were transcribed using GoodTapes. The data material was analyzed using the data software Nvivo, and transcribed interviews and written observations were coded thematically (Braun and Clarke 2006) with a primary focus on situated practices of digital support and social settings within the three aforementioned forms of digital support (formal, informal, and nonformal). Based on the coded data material, statements and field notes were collectively analyzed and discussed by the authors in workshops and meetings. Furthermore, these results were presented and discussed with stakeholders and municipal partners.

All interlocutors have been anonymized or pseudonymized, balancing to maintain the contexts of digital support while not disclosing their identities or location. As digital support was often carried out and received in relatively safe spaces, some supporting and supported persons may be able to recognize each other’s statements in the following examples. All interlocutors have been knowledgeable about the field work that was carried out in their sites of digital support. The semi-structured interviews were conducted with written and informed consent, and observations and informal conversations were conducted with informed oral consent. All data materials are securely stored according to university guidelines.

### New materialist response-ability

Inspired by new materialist-feminist discussions, our analysis draws on the notion of distributed agency as described by Jane Bennett’s (2010; 2012; 2005; 2004) and Donna Haraway’s (2016; 2007) reconceptualization of responsibility as response-ability. In different ways, Bennett and Haraway address the radical and reciprocal relationalities at play between humans and non-humans—“things” and animals, respectively—and argue that nothing comes before relating, i.e., that we cannot understand or study humans or things without understanding their relational nature. The relation is the smallest analytical category, not the entity itself, as this category only exists through its relations (Haraway 2003, 12). In our analysis, we emphasize how computers, smartphones, printers, Wi-Fi, digital platforms, communications, and other people help, support, and guide Danish citizens’ ability to navigate and respond to digital means of communication. We thus argue for a greater focus on the many agents (human, material, digital) that make digital citizen-state relations possible.

Bennett argues that agency is the effect of relations of heterogeneous actants within assemblages, and she draws on the vocabulary and theoretical interest of work within the fields of Science and Technology Studies and Actor-Network Theory (Latour 1996; Latour and Woolgar 1986; Law 2002; Callon 1990; Mol 2002; Barad 2007; Haraway 2000; 2003). However, Bennett accentuates the agency of “things” and aligns it with human agency. Drawing on Latour, she emphasizes the role of actants as both sources of action and as “interveners” (Bennett 2010, 9), highlighting how actants (all “bodies”) are swarms of “small agencies” always in “competition or confederation with many other strivings” (Bennett 2010, 32). Bennett’s theory of distributed agency highlights that assemblages owe their agentic capacities to the materials that constitute them. With an analogy to the Chinese concept of “shi,” agency is understood as forms of energy that does not originate in human initiative but is the result of the disposition of things (Bennett 2010, 35). Humans too may have a will and an intention, but human action must be understood in relation to the many other currents that follow, affect, and hinder its path. In the following analysis, we draw an opposition between the intervening and responsive capacity of actants. The capacities to respond and intervene reside within the assemblages where the affecting/affected actant finds itself. Herein, we argue, may lie what Haraway calls response-ability: an ability to respond that is an effect of the assemblage in question:

[M]attering is always inside connections that demand and enable response, not bare calculation or ranking. Response, of course, grows with the capacity to respond, that is, responsibility. Such a capacity can be shaped only in and for multidirectional relationships, in which always more than one responsive entity is in the process of becoming. (Haraway 2007, 71)

Asking how to become able to respond situates the doer in complex relations and relieves the doer from solitary autonomy. In continuously asking how to become able to respond, we seek to critically investigate the agency of digital citizens, asking, as Bennett: “Perhaps the responsibility of individual humans may reside most significantly in one’s response to the assemblages in which one finds oneself participating” (Bennett 2005, 464).

In the first part of our analysis, we introduce the assemblages of distributed intervening: citizens and their connection to the *public digital*. We argue that this connection mediates a distributed agency of many composite actants—devices, printers, self-service platforms, digital mail, et cetera—that demands the digital response of citizens. In the second part, we focus on examples of older adults needing digital support, and we argue that the support must be seen as responses mitigating the public digital intervention in daily life. These responses are socio-materially entangled and distributed across human and non-human actants.

### The public digital as distributed intervening: Material changes, composite actants, and mediated intervening

Back then, [tax returns] were a cumbersome process. You had to fill out forms, and you had to write a lot of different tax returns and stuff like that. It was very cumbersome for the average citizen but very understandable. You knew what you were doing. It was difficult to do a tax return, but very understandable because you could see what was happening. You can compare it to the typewriter, which you can see going up. A ‘D,’ it came down, boom, and there was a clear connection. When you type on a keyboard for a text message it corrects the spelling. No one can see where it’s coming from. No one can figure out where it is. So, it matters that you understand what's happening. (Jan)

In the quote, Jan, 78, tells of his experience with the material changes of technology. He makes a rough distinction between the cumbersome but somewhat logical materiality of on-paper tax forms and the digital ease and efficiency, but inaccessible materiality, of the connections within the technologies of a keyboard and devices that auto-correct your spelling. This transition can be experienced as both black boxing and a loss of connection if you do not understand its workings (Latour 1987; Löfgren 2014). In this part, we argue that the public digital, experienced as a ubiquitous totality, is assemblages that *intervene* in citizens’ lives in distributed ways. Here, we highlight the material changes of public digital intervention, the composite actants that the interventions rely on, and the mediated forms of agencies that intervene.

### Material changes

The older adult interlocutors in this study have witnessed firsthand the transformation from on-paper formulas and face-to-face encounters with public authorities to the increasingly digital self-service of public administration in the last two decades. Accumulated experiences and know-how of one materiality of bureaucracy have become of little value as the materialities, and consequently, logics change (Rønning and Sølvberg 2020).

Transitioning from physical to digital mail shows a material change similar to Jan’s description. In the following quote, a public healthcare professional tells us of her conversations with older adults when visiting them on preventive home visits.^1^ The purpose of these meetings is to begin preventive conversations on health and well-being and to inform about services from the authorities and other organizations. In advance, the municipality notifies the citizen of the visit through public digital mail. Therefore, when the healthcare professional visits, it becomes apparent whether the citizen has seen this digital mail. This can, according to the healthcare professional, lead to conversations about senior citizens’ digital usage:

When we visit them and they say: ‘We didn’t see that, I haven’t received a letter,’ ‘but do you have digital mail?’ ‘Yes,’ ‘but have you been online to have a look?’ And then sometimes they let us help them log in and look, and they can see that the letter is there. And it’s dated such and such, and then we realize that there’s also something else; there’s something from the bank and there’s.... ‘Well, I’d better have a look at that,’ because they don’t go online and look every day like they did when they had the mailbox. They don’t do that. But we can also guide them to receive a text message when there’s something new in their digital mail. (Healthcare professional)

The healthcare professional describes how she helps a citizen with logging on to their digital mail provider. But the quote also tells of a material change, as public communication about health as well as other issues now by default will be digital and not sent to the citizen’s physical mailbox. New routines and strategies involving new materialities are due. But she also points to an opportunity that grants the senior citizen a new digitized response-ability. She suggests that the citizen should agree to receive a text message that replaces the physicality of the everyday check of the mailbox. Checking public digital mail then becomes reliant on a text message notification, reminding the citizen to check the digital mailbox, but this solution also requires several necessary digital skills.

This is a material change through which new distributed assemblages of public digital intervening are introduced—the importance of checking one’s mail has not changed, but new material expectations have changed the citizens’ routines. The materialities of public digital communication and interactions—the papers, smartphones, two-factor authenticators, internet access, and keyboards—all have small agencies that inform this assemblage. These materialities apply constraints and possibilities to the citizen’s digital interaction and encounter with authorities and healthcare professionals,^2^ etc. The diverse actants and agencies are either in confederation or in competition with the citizens (depending on skills, know-how, et cetera) and cultivate different demands of response-ability (Haraway 2016, 132). The ability to respond to intervening agencies of the digital public sector is a matter of the citizen relating to and working with material-digital mediators of the welfare state.

Public digital response-ability is ongoing and a matter of reciprocal processes of becomings of assemblages that configure citizens, state, and the mediating hardware and digital platforms. As the older adults we interviewed have been part of other assemblages of citizen response-ability during their lives, they possess other literacies and know-how formed through human and non-human entanglements of “pre-digital” materialities of welfare response, which now seem outdated.

### Composite actants

All digital and technical actants are always composite, and the assemblages of the public digital work *through* a set of composite actants. Accessing public digital mail or login via two-factor authentication requires functional computers, smartphones, and Wi-Fi access. A municipality-employed librarian in charge of a digital support café explains how these various technical and bureaucratic requirements can cause a lot of confusion:

Many of the people buying a new phone also want a new passport because it’s been communicated that you need a phone that can do this and to get that one, you need a passport that’s not too old …. But there are also many people with a computer that’s over 10 years old. We can hardly do anything if they don’t get a new computer. Many people are having problems with digital mail, MitID, Borger.dk^3^ …, and they must have a device they can use and rely on, so they get their messages. And with some people, you just think to yourself: ‘Okay, listen, I think you should be exempt from the digital’ but it’s always a last resort. (Librarian)

The quote is quite dense: To be able to use MitID, the public two-factor login authenticator, on your phone, you need to register. To register from home, you need a passport that has a built-in chip, and you need a smartphone that is able to read this. Consequently, if your smartphone or passport is too old, you cannot register. Additionally, your computer cannot be too old to operate public digital websites. The state of your devices matters for your ability to interact and respond to public digital communication, as doing anything online requires reliable technology. Being able to respond digitally then relies on a large set of composite actants, i.e., a working computer, smartphone, and passport that arguably are all assemblages on their own.

The newness of your passport and the reliability of your smartphone and computer are just a few of many smaller agencies whose accumulated effects make up the citizen’s agency. The ability to respond is a matter of being able to work *with* these sets of composite actants.

### Mediated intervening

Jan told us of a relative’s passing and of interacting with the probate court, e.g., uploading the right documents with the right information to the right probate court. While being emotionally affected by his loss, he also had to find the right district of the probate court and report the relative’s death. He had to make sure that he had the power of attorney to do so, he had to navigate the digital submission system, and he had to make sure that he had all the right information. When we asked him where he looked for help, he hesitated. The municipal service centers and local digital cafés at the library were not an option. He had tried them before but felt that his issues were too specific. “What about asking your children?” we asked. He thought about it for a while and said:

Why don’t I promptly answer ‘yes’? I think it has to do with the fact that if I have a problem handling my relative’s death, I can’t go to my children because they’ve never been in that situation. I talk to friends and acquaintances. … I have some friends who feel the same way. If my mom died, or if my uncle died, they’re the ones I talk to. I think the answer is that I go to someone in my age group who has been in the same situations. And that’s because the problem is not about whether they have insight into the digitalization of the phone. It’s about them having insight into the life situation I’m in.

Jan’s situation is simultaneously a personal problem and a socio-technical problem, as it regards seeking out the proper choices within the digitalized bureaucracy of the probate court. Jan describes how his digitalized administrative obligations as a family member (and citizen) rely on talking with peers with similar experiences, sharing the know-how of complicated interfaces, and attending to life situations. Jan’s story tells of these entanglements because the death of his relative required interrelated actions of him as an affected and social human being and as an administrative actor entangled in timely digital actions via a computer and uploading documents with the right information. The public digital required a response from him and, mediated through his devices, intervened in his grief by demanding his agency.

The agency of the public digital as an assemblage is agentic in mediated and mediating forms through composite actants. On one end, the public digital is compounded of various interfaces that facilitate communication between an (often automated) authority following protocol, keeping up with laws and rules, and deadlines and consequences (e.g., fiscal and access to healthcare). On the other end is a citizen who must respond to a digital self-service interface mediating a digital-authority-law-technical assemblage. In and between this is a range of materialities (computers, smartphones, tablets, Wi-Fi, printers, etc.) that mediate and assist both the intervening and the response.

In the following, Mikkel, an IT educator at a senior housing area, explains how notifications and pop-ups that appear on devices may have intervening and disrupting effects. This is agencies of the digital that prompt a reaction and require a certain ability to respond:

It’s the tiny little things that can go wrong. They may have pressed twice on their phone instead of once so that the browser closes instead of opening. It’s those micro things that put them completely off course. And there are a lot of people who have asked for manuals, or video manuals, where I show them how to do it. The problem is just that ... when something pops up like; ‘We can see that this browser hasn’t been updated, do you want to update?’ and stuff like that. Or it has changed its appearance, or an update has been made that changes something. ... You can say a lot of bad things about MitID, but they’ve really done everything they could, I feel, to make it user-friendly. I mean, it literally says: ‘Now open the app,’ but if you don’t understand that you’re in one app—your browser—and that you have to go and open another app, then you can’t use it.

Mikkel emphasizes how various *things*—materialities and pop-ups—might set one off course. The citizen may be asked by the device, website, or app to do a certain thing and that depends on the citizen’s ability to understand and act on that request. The IT educator exemplifies this with the MitID app, which requires citizens to be able to switch between apps on their smartphones. Sharing experiences or getting advice and help may be needed. In the following, we explore how responding digitally also has to be understood as socio-material sites that distribute the practices of response among several actors.

## Digital support as distributed response: Socio-material sites, proximate alliances, and safety-courtesy choreographies

Sites of digital support are socio-material sites, where citizens’ public digital issues become a continuous relational matter. During our ethnographic fieldwork, we found that public digitalization is entangled in all sorts of social situations, where citizens help each other, exchange experiences and know-how of specific digital doings, consult each other on bureaucratic formulas and choices, or help set up. Sites of digital support stand out as a contradiction to the national narrative depicting citizens sitting by themselves in front of a computer handling their digital affairs on their own. In contrast, we argue that seeking support is a fundamental aspect of digital practices.^4^

The following analysis is primarily based on field notes from sites of digital support, where older adults find help and guidance when experiencing difficulties independently interacting with public digital services. As such, we argue that digital support relies on the continuity within social sites, proximate and co-constituting alliances, and that digital support involves situated and local safety-courtesy choreographies. The ability to respond lies within supporting socio-material settings that make citizens able to respond materially, digitally, and socially.

### Social sites of digital support

Fieldnote 1: We enter a small café in a major Danish city. The windows are covered with curtains hanging from the middle of big and wide windows, a classic interior design of older bodegas and inns. For a lack of better terms, this is a community café that offers a place to be, socialize with peers, and combat loneliness among older adults. As we enter, volunteers make things ready, brew coffee, and prepare traditional open sandwiches on rye bread. This is a place meant for socializing. Senior citizens come to chat, to get lunch and a cup of coffee, sometimes there are activities and events, but it has also grown into a place, where guests find help, guidance, and support on digital matters. As we talk to guests and volunteers, they point to the community center manager as the primary digital support person. On the communal computer, the manager supports the guests in various ways, from ordering train tickets online, setting up MitID, or reading documents sent by the authorities. And, crucially, the communal computer is connected to wifi, a printer, and a scanner. Having access to a printer and scanner can be essential when sending or signing legal documents.

One woman at the community café tells us that she ‘hangs in with her nails’ as she struggles to keep up with the digital development, but she tries and wants to be independent for as long as possible. But when her tablet needs an update or she faces issues with her MitID, she reaches out to the community center manager. At her home, she also often gets help from a person she calls the deputy chairman, referring to this person’s role in the residential complex. The woman explains that she also gets help from her daughter, but she’s not always around, and the manager and the deputy chairman are both conveniently accessible at the two places where she spends most of her time. ‘If you have two or three to help you,’ she says, ‘that should do it.’

The manager is praised for her flexibility, patience, and pedagogical skills, as well as for being trustworthy and reliable. She has been around for 12 years, and the regular guests know her well. A combination of the willingness and interest of the manager to help, the shared needs of the guests, and the communal computer has made the community center a place for digital support. As guests have lunch and join social activities, trusting relations are building. Digital support becomes convenient as they are already here. For some guests, in turn, the digital support becomes the reason to come here, as it becomes the place to manage digital errands such as communicating with the healthcare system.

In this example, the ability to respond to tablet updates or public digital requests is distributed among trusted digital supporters. In our dialogue with the woman at the café, she explains that she can handle most of her digital affairs. But when something new or challenging arises, the community center and its manager are close by and ready to help her navigate, enable her decision-making, and perform the tasks. Solving digital issues becomes a continuous relational matter.

### Proximate alliances

Field note 2: In the function room of a common house in a senior citizens’ housing area, Mikkel organizes a weekly digital café for the residents. A pool table is covered, and behind a column, a printer is on a table. As we greet Mikkel, he has already prepared one table with coffee cups, small plates, and napkins. At 10 o’clock, the first group of residents enter the room and finds a seat. Routinely, everyone has brought along either a tablet or a computer. Two women start playing games on their tablets, sometimes showing progress to each other. One bursts out that now her tablet suddenly works, as it didn’t earlier in her apartment, ‘well, that was a delight.’ Others wait for Mikkel to help them as he goes around helping one, then the next. While they come here for digital support, the socializing and the coffee break at 11 o’clock are also stressed.

When interviewing Mikkel, he explains that it is the day-to-day problems that are most common. How to transfer money, how to access public digital mail through e-Boks,^5^ or how to fill out a form before a doctor’s appointment. Having done this for five years every week, Mikkel has become familiar with most of the residents. A community has been built within this weekly time and space, where the residents repeatedly come to socialize and, in a safe space, get digital support.

Jane, 75, attends the digital café every week. She praises Mikkel for helping her become digital and acknowledges that she needs continued support from others to maintain it. She explains:

I think we’re very fortunate here that we can get help in this way. And that’s also why you keep coming back here. Because Mikkel has the patience of an angel and because you forget when you don’t use it all the time. We would never have managed MitID if we didn’t have Mikkel. Then next week he comes back and says: ‘Well, show me [that you can log in to] your MitID.’ And that’s good because we don’t use it the way you [younger generations; the interviewer] use it.’

In the quote, Jane emphasizes that she *is* digital by her own measurements.^6^ She uses her MitID and various public digital platforms, but she knows that this use relies on an amount of continued support. As we ask about her use of e-Boks, she replies: ‘Yes, I use that [e-Boks]. But that’s only because Mikkel has shown me 140 times. Otherwise, I wouldn’t have dared. And as I told you, he is so patient and then we do it one more time and one more.’

Mikkel gets high appraisals for his pedagogical skills and patience as an educator and guide by the older adults attending the café. Learning environments can take many forms, and other studies show that social support can be crucial in helping older adults become acquainted with digitalized functions (Kuoppamäki et al. 2022; Tsai et al. 2017). A Norwegian study on arenas for digital learning has described digital competencies as “fresh produce,” as digital competencies quickly become outdated (Rønning and Sølvberg 2020). Following this analogy, Mikkel’s continued digital support helps the residents’ digital competencies stay fresh while highlighting that digital competence is a continuously co-constituted doing—and not a “having.” Accordingly, as the assemblage of the public digital intervenes and demands Jane’s response, Mikkel’s continued support co-constitutes her response-ability.

The resident’s digital presence depends on the support from Mikkel and others, but importantly, this is an alliance that seeks to keep them as autonomous as possible and promote decision-making and self-determination within the public digital bureaucratic doings. As suggested by Carreras and Finken (2022), these forms of “autonomy alliances” reconfigure on-screen services as social situations wherein public digital matters can be discussed, weighed, and better understood. Digital support involves not only the support, help, or guidance on a specific digital doing (e.g., access to healthcare) but also addresses and makes sense of the wider material assemblage that the digital doing is part of.

### Safety-courtesy choreographies

Continuing field note 2: Mikkel sits down with a woman having trouble logging into e-Boks. Mikkel has previously helped her write down her passwords and the related web addresses on a piece of paper and Mikkel now points to this overview, as she has to log in using MitID. First, she has to write her username on the browser log-in page, then she has to confirm on the MitID app. ‘Now you need your password,’ Mikkel says, ‘and that’s secret, but remember you have written it down.’ She looks at the password on the paper as she writes the password into the app and then she has to swipe.

By Danish law on MitID, individual passwords are not to be shared. As Mikkel, aware of this, experiences that residents need help remembering their passwords, Mikkel suggests that they write down their passwords. It is an analog helper in the form of a piece of paper containing information that, by Danish law, is considered strictly personal. As Mikkel has supervised her log-in—and has helped her write the password on paper—it is by authorities considered a misuse. The piece of paper ultimately presents a small, material, and unwillingly illicit agency that affects the residents’ everyday use of public digital platforms.

In a different location and city, we meet the social worker Agnete, in a social housing area. She works with supporting the residents and organizes weekly open office hours, where they can come and get support regarding their communication and interaction with the public authorities, typically focusing on the public digital self-service platforms. Agnete tells us that to get her help, the residents have to have their MitID in order:

We can’t help anyone with anything unless they have either NemID^7^ or MitID with them. We sit with the computer between us and turn the screen [back and forth]: now you have to enter your CPR number, now you have to enter your code. Often we turn the screen and they press their password, then they enter their e-Boks and then they have to log off themselves. I think that can be a bit sensitive in this guidance work, because if you’re not very digitally skilled, do you think about the consequences of leaving your things open?

To get support from Agnete, residents need their two-factor login authenticator, MitID, or to have the prerequisites to get help obtaining it. Agnete then evokes a three-part safety-courtesy choreography of information disclosure that involves Agnete, the resident, and the turning computer center stage, wherein the supported resident’s privacy writing their password and log-in is enacted. After logging in, they can attend to the matter at hand, and at the end, the resident can log off on their own. It is a safety and courtesy measure that she upholds to keep residents’ private information undisclosed to her and at a legal distance. She elaborates on this safety-courtesy choreography:

One Friday, just before closing, someone said: ‘Can you help me with a three-month bank statement from my bank?’ I turn the computer around and he logs on to online banking. I then look because I need to see that he checks the right boxes so we get three months on the paper statement and then I say that I’m not looking afterwards, I just turn around, ‘I haven’t read where you’ve been shopping.’ And then I tell him to press log off, so we can log off. ... What if someone suspects you of seeing something you shouldn’t have seen? But it’s that thing about being a guiding hand at their back. He could have gone to the citizen service or the library and borrowed a computer and printer himself, but it is to help navigate through the confusion because we don’t have passwords and would never be able to go to that page afterward. ... But I do feel that it's sensitive for him and also for myself. But then we press send and then he gets it in paper in a plastic pocket and walks off with it.

The safety-courtesy choreography has multiple steps and changing digital and material partners; an additional step of logging into online banking, which is not mentioned in the quote, is the MitID two-factor login authentication that requires the resident’s smartphone. The resident enters his online banking platform, where Agnete takes a look to make sure he checks the right boxes and then makes it explicit in words and body language that she will not peek at the resident’s spending. Then they print the account statements, put them in a plastic pocket, and she makes sure that he logs off the computer. The safety-courtesy choreography is maintained and structured by Agnete, who is very aware of the confidentiality of her support and wants to minimize the amount of personal information disclosed to her. The choreography is then, in other words, formed by the needs of the resident, wherein he implicitly consents to a degree of disclosure of information and the explicit ways Agnete structures the situation of support to signal orally and bodily that she is not interested in his personal information.

Agnete’s safety-courtesy choreography—her way of practicing digital support—is different from earlier examples in this article. The IT educator, Mikkel, who helps residents write their passwords down on paper, is an example of a different choreography, with differently situated negotiations of safety and courtesy. It is relational and socio-material choreographies that in coordination seek to make the supported person able to respond to a required digital doing. They may be situational and different in practice but regard the same demanding agencies and effects of public digital self-service platforms, mail, and two-factor authentication login through devices and computers with internet access. When public digital development is seen as a physical obstacle, digital support mitigates these challenges. It initiates new alliances of humans and materialities to collectively respond to the individualizing demands of the digital welfare state.

## Medical humanities and the search for harmful effects

Digital support is practiced in various forms, and, as we have argued, practices of digital support are collective and distributed responses to mediated bureaucratic interventions of the assemblage of the public digital. While the Danish politics of digitalization place the responsibility to respond to public digital demands on the citizen, we have presented how citizens’ response-ability is the result of a range of small material and digital agencies that do not always align. Digital support, then, is distributed ways of mitigating and responding when the public requires the response of the citizens despite their lacking response-ability.

In this article, we have sought to highlight the heterogeneous and ever-changing prerequisites of being a Danish digital citizen capable of autonomously interacting with the digitalized welfare state. What is at odds is that while citizens are politically upheld as individual and self-reliant digital agents, these agentic capacities are the result of a non-static list of human, material, and digital actants and smaller agencies that make citizens response-able. Consequently, response-abilities as forms of agency have to be understood as constituted by a distributed set of actants, in the context of this article, that involve social relations, authorities, healthcare professionals, public employees, hardware (devices, keyboards, printers, scanners, Wi-Fi-connection), software, public digital platforms, log-in systems, and public digital mail distribution, and so on.

Because of the individualizing logics that dominate the Danish public digital developments, digital support has an illicit and suspicious legal status. Therefore, our findings tell of the limitations of current Danish digital regulations that do not fit real-world digital practices. The many examples of digital support we witnessed were always done with clear consent, within safe spaces, and to maintain citizens’ digital autonomy (see also Carreras and Finken 2022). This could be the necessary general sharing of personal information within public digital platforms and formulas or the practices of assisting in generating or sharing passwords to the publicly distributed two-factor authenticator log-in, MitID, in situations of support, which is explicitly stated as misuse by law.

We end this article with two final and related notes on the role of medical humanities, and on individual responsibility. As medical humanities has been traditionally concerned with the patient-doctor relation of the medical encounter, public digitalization in the Nordic welfare states arguably regards both the before, during, and after of the medical humanities “primal scene” of the clinical encounter (Whitehead and Woods 2016). In addition, it has been argued that medical humanities should engage with how health, illness, and treatment “are constituted in and through tangled webs of human and non-human biosocial organisms, political-economic formations, discourses and affects” (Viney et al. 2015, 2). Here, we may add the tangled webs of material-digital accessibility of patients and citizens, as digitalization and technological developments have become part of all medical encounters—for good and bad (Rampton et al. 2022). Therefore, medical humanities should be increasingly concerned with the challenges digitalization imposes on population groups when made vulnerable by public digital demands of digital response-ability. As digitalization tends to generate an increasingly individualizing gaze, medical humanities must be critically concerned with the manifold, subtle actants that co-constitute accessibility and responsiveness of patients and citizens.

Secondly, an informed, critical gaze should always address normative statements of responsibility concerning digitalized access to welfare. As the Danish welfare consists of evermore heterogeneous and dynamic assemblages of human, material, and digital actants that span the physical and digital spheres, a singular blame game becomes increasingly problematic. Bennett’s suggestion then, paraphrasing Hannah Arendt, is to look for “sources of harmful effects” (Bennett 2010, 37) within complex assemblages. Crucially, digital developments are not the villain, but there are sources of harmful effects within assemblages of public digitalization in Denmark and elsewhere that are of interest to researchers of medical humanities that seek to address how we culturally and socially experience health and medicine in an increasingly digitalized world.

## Data Availability

The data is not in a public depository as participants have been promised anonymity and anonymizing raw data completely is impossible with this kind of data.

## Declarations

### Endnotes

^1^ Preventive home visits are nationwide services provided by local municipal healthcare professionals in case of the death of a spouse, health, mental, or social concerns, or yearly after citizens turn 80 years old. With the recent Ældrereform (“Seniors reform”), municipalities will no longer be required to provide this service.

^2^ For a historical analysis of technologies role within patient-physician relationships, see also Rampton, Böhmer, and Winkler (2022).

^3^ Translated as citizen.dk, borger.dk is the primary platform for municipal or welfare state inquiries.

^4^ For a Danish elaboration of this argument, see Andersen et al. (2024).

^5^ This is one platform for accessing public digital mail.

^6^ Importantly, digital usage is not an either-or, but processual and fluid. Research in Socio-Gerontechnology has highlighted this (Gallistl and Wanka 2022; Gallistl et al. 2021; Rosales and Fernández-Ardèvol 2020).

^7^ NemID (EasyID) is the precursor to the current two-factor login authenticator MitID (MyID).
